# A single center evaluation of applicant experiences in virtual interviews across eight internal medicine subspecialty fellowship programs

**DOI:** 10.1080/10872981.2021.1946237

**Published:** 2021-06-30

**Authors:** Laura A. Huppert, Gerald Hsu, Najwa Elnachef, Lynn Flint, James A. Frank, Lianne S. Gensler, Edward C. Hsiao, Raman R. Khanna, Atif Qasim, Brian S. Schwartz, Eric Widera, Carly Zapata, Jennifer M. Babik

**Affiliations:** a Hematology/Oncology Fellow in the Hematology/Oncology Division in the Department of Medicine at the University of California, San Francisco, CA, USA; b Associate Professor in the Hematology/Oncology Division in the Department of Medicine and Program Director of the Hematology/Oncology Fellowship Program at the University of California, San Francisco, CA, USA; c Associate Professor in the Gastroenterology Division in the Department of Medicine and Program Director of the Gastroenterology Fellowship at the University of California, San Francisco, CA, USA; d Associate Professor in the Division of Geriatrics in the Department of Medicine and Program Director of the Integrated Geriatrics and Palliative Care Medicine Fellowship at the University of California, San Francisco, CA, USA; e Professor in the Pulmonary/Critical Care Division in the Department of Medicine and Program Director of the Pulmonary and Critical Care Fellowship at the University of California San Francisco and San Francisco VA Medical Center, San Francisco, CA, USA; f Professor in the Rheumatology Division and Russell/Engleman Rheumatology Research Center in the Department of Medicine and Program Director of the Rheumatology Fellowship Program at the University of California, San Francisco, CA, USA; g Associate Professor in the Division of Endocrinology, Department of Medicine, and Program Director of the Diabetes, Endocrinology, and Metabolism Fellowship Program at the University of California, San Francisco, CA, USA; h Division of Hospital Medicine and Program Director of the Clinical Informatics Fellowship Program at the University of California, San Francisco, CA, USA; i Associate Professor in the Cardiology Division in the Department of Medicine and Program Director of the Cardiology Fellowship at the University of California, San Francisco, CA, USA; j Professor in the Infectious Diseases Division in the Department of Medicine and Program Director of the Infectious Diseases Fellowship at the University of California, San Francisco, CA, USA; k Professor in the Division of Geriatrics in the Department of Medicine and Program Director of the Geriatric Medicine Fellowship at the University of California, San Francisco, CA, USA; l Assistant Professor in the Hospice and Palliative Care Medicine Division in the Department of Medicine and Program Director of the Hospice and Palliative Medicine Fellowship at the University of California, San Francisco, CA, USA; m Associate Professor in the Infectious Disease Division in the Department of Medicine, Associate Program Director of the Internal Medicine Residency, and Associate Program Director of the Infectious Disease Fellowship at the University of California, San Francisco, CA, USA

**Keywords:** Virtual interview, remote interviews, fellowship interviews, covid-19

## Abstract

Due to the COVID-19 pandemic, most graduate medical education (GME) training programs conducted virtual interviews for prospective trainees during the 2020–2021 application cycle. Many internal medicine (IM) subspecialty fellowship programs hosted virtual interviews for the first time with little published data to guide best practices.

To evaluate how IM subspecialty fellowship applicants perceived the virtual interview day experience.

We designed a 38-item questionnaire that was sent via email to applicants in eight IM subspecialty programs at a single tertiary academic medical center (University of California, San Francisco) from September–November, 2020.

Seventy-five applicants completed the survey (75/244, 30.7%), including applicants from all eight fellowship programs. Most survey respondents agreed that the length of the virtual interview day (mean = 6.4 hours) was long enough to gather the information they needed (n = 65, 86.7%) and short enough to prevent fatigue (n = 55, 73.3%). Almost all survey respondents agreed that they could adequately assess the clinical experience (n = 71, 97.3%), research opportunities (n = 72, 98.6%), and program culture (n = 68, 93.2%). Of the respondents who attended a virtual educational conference, most agreed it helped to provide a sense of the program’s educational culture (n = 20, 66.7%). Areas for improvement were identified, with some survey respondents reporting that the virtual interview day was too long (n = 11) or that they would have preferred to meet more fellows (n = 10).

Survey respondents indicated that the virtual interview was an adequate format to learn about fellowship programs. These findings can inform future virtual interviews for GME training programs.

## Introduction

Due to the COVID-19 pandemic, graduate medical education (GME) training programs conducted interviews for prospective trainees using a virtual format for the 2020–2021 application cycle[1]. Many internal medicine (IM) subspecialty fellowship programs hosted virtual interviews for the first time with little published data to guide best practices.

Interviews are an important component of the subspecialty fellowship application process, often affecting the final rank order of both programs and applicants [2,3]. Previously, most GME training programs conducted in-person interviews at the site(s) of the training program[4], A small number of programs previously reported their experiences with virtual interviews [4–11]. In these studies, applicants reported that they were able to present themselves to their satisfaction [10,11], gain a satisfactory understanding of the program[10], ask questions of fellows and faculty[11], and had an overall positive virtual interview experience [8,11]. In contrast, some applicants reported that virtual interviews were less effective than an on-site interview[6], indicated a preference for interviewing in person[11], or reported that the virtual interview experience had an unfavorable impact on their rank position of the program[10], although reasons for these views were not fully explored. However, these were all studies from single fellowship programs with a small number of respondents and they did not evaluate specific aspects of the virtual interview day (e.g., the optimal number and length of interviews, how to structure interactions with fellows, whether or not to have applicants observe didactics, etc.). Moreover, as most studies did not report the demographic data of the applicants, it was not known whether the experience differed among demographic groups. As such, best practices for constructing a virtual interview day are unknown.

We aimed to address this gap by conducting a comprehensive, multi-program evaluation of the virtual interview applicant experience at our institution. We surveyed applicants from eight internal medicine (IM) subspecialty fellowship programs about the elements and effectiveness of the virtual interview day, allowing for comparisons between interview structures and fellowships.

## Methods

### Setting and participants

Eight IM subspecialty fellowship programs at the University of California, San Francisco (UCSF) participated in this study: clinical informatics, endocrinology, gastroenterology, geriatrics, hematology/oncology, hospice and palliative medicine, infectious diseases, and rheumatology. Each of these 8 programs conducted only virtual interviews, with no in-person interviews offered to any applicant. Features of the planned virtual interview day for each fellowship program are shown in Supplementary Table 1. Fellowship applicants from all eight programs (n = 244) were emailed an anonymous survey link on the day they completed their UCSF fellowship interview. Participation was voluntary, and no incentives were provided. The email and survey text explicitly stated that the survey results would not be examined until after the National Fellowship Resident Matching Program (NRMP) Match Day. Survey responses were collected between September and November, 2020.

**Table 1.** Demographic information about survey respondents

	**Self-reported** **demographic data (n = 75)** n (% survey respondents)
**Gender**	
Female	33 (44.0%)
Male	35 (46.7%)
Non-binary	1 (1.3%)
Prefer not to answer	1 (1.3%)
No response^a^	5 (6.7%)
**Current year in training**	
Second year resident (PGY2)	1 (1.3%)
Third year resident (PGY3)	48 (64.0%)
One year post residency	12 (16.0%)
Two years post residency	0 (0%)
Three or more years post residency	7 (9.3%)
Prefer not to answer	2 (2.7%)
No response^a^	5 (6.7%)
**Race/Ethnicity (select all that apply)**	
Hispanic, Latino, or Spanish origin	7 (9.3%)
American Indian or Alaskan Native	0 (0%)
Asian	19 (25.3%)
Black or African American	6 (24.0%)
Native Hawaiian or Pacific Islander	2 (2.7%)
White	34 (45.3%)
Prefer not to answer	4 (5.3%)
No response^a^	5 (6.7%)
**Underrepresented in Medicine (UIM)**^b^	
UIM	15 (20.0%)
Not UIM	51 (68.0%)
Prefer not to answer	4 (5.3%)
No response^a^	5 (6.7%)
**Fellowship Program**	
Clinical informatics	7 (9.3%)
Endocrinology	7 (9.3%)
Gastroenterology	7 (9.3%)
Geriatrics^c^	4 (5.3%)
Hematology/oncology	22 (29.3%)
Hospice and palliative medicine	7 (9.3%)
Infectious Diseases	14 (18.7%)
Rheumatology	2 (2.7%)
No response^a^	5 (6.7%)

^a^Survey respondents who did not respond to the demographic data were labeled as ‘no response’.

^b^See methods section for definition and inclusion criteria for UIM

^c^Geriatrics/palliative combined fellowship included in the Geriatrics fellowship respondents

### Survey design and outcomes measured

We designed a 38-item questionnaire with Qualtrics Survey Software (Provo, UT; Seattle, WA) using Artino’s survey design process[12]. See full survey instrument in Supplemental Data. The survey included quantitative and qualitative sections. Item types included multiple choice, sliding scale, 5-point Likert scale, and open-ended questions. Both complete (70) and incomplete (5) responses were included in calculating the response rate, and questions that were answered on incomplete surveys were included in the data analysis.

### Demographic data definitions

We used the Association of American Medical Colleges (AAMC) definition of Underrepresented in Medicine (UIM)[13], which is defined as individuals who self-identify as African American or Black, Hispanic or Latino, American Indian or Alaska Native, or Native Hawaiian or Pacific Islander[14].

### Data analysis

Quantitative data were analyzed as received, including entries that may have been omitted, in order to maintain data integrity. For Likert scale questions, ‘strongly agree’ and ‘somewhat agree’ were considered favorable responses (combined as ‘agree’ throughout the results section) and ‘neither agree nor disagree’, ‘somewhat disagree’, and ‘strongly disagree’ were considered unfavorable responses. Data was analyzed using Prism Software (GraphPad; San Diego, CA). We used descriptive statistics to summarize numeric responses. Comparisons between groups were made using the unpaired t-test or two-sided Fisher’s exact test, as appropriate. A p value of <0.05 was considered statistically significant.

Qualitative survey data was analyzed using inductive content analysis to identify themes[15]. One investigator (LAH) coded all data and two other investigators (GH, JMB) each reviewed 50% of the coded responses for agreement. Differences in coding were discussed and reconciled.

### IRB statement

The study was deemed exempt by the UCSF Institutional Review Board.

## Results

### Survey respondent characteristics

We invited 244 applicants from eight IM subspecialty fellowship programs to participate. 75 applicants responded, yielding an overall response rate of 30.7% (Table 1). Of the 75 respondents, 33 identified as female (44.0%) and most were in their third year of residency training (n = 48, 64.0%). 15 respondents self-identified as UIM (20.0%). The survey response rate varied by subspecialty (Supplementary Table 2). Demographics of survey respondents vs. non-respondents are shown in Supplementary Table 3.

**Table 2.** Overall strengths and weaknesses of the virtual interview day experience: Results from content analysis of responses to open-ended items

**Domain**	**Strengths**^a^	**Weaknesses/Areas for improvement**^a^
Structure/organization of the virtual interview day	‘I liked the dedicated overview of the program at the beginning (better than if it was a recorded video).’ ‘It was well structured, there were enough breaks, the tip sheet beforehand was very helpful.’ ‘Appropriate length of individual interviews and overall day. The supplemental videos provided introducing applicants to the hospital and various resources were very helpful.’ ‘I very much appreciated the 15 minute breaks (as opposed to 5 min breaks or no breaks) between interview sessions and found these to be crucial.’ ‘The technical support was strong and I knew I could reach out to the program coordinator.’ ‘The virtual format allowed me to save time and money.’	‘The approach of having one general “waiting room” for all of the fellows and then sending us in and out of breakout rooms was not a good experience … There were often delays in sending us to our breakout rooms, which added to the fatigue of the day.’ ‘I would suggest making it clear that lunch time is for eating. It wasn’t clear, so we ended up having 10 minutes at the end of the “fellows lunch” period to eat something.’ ‘I think that it would have been really beneficial to send a packet or PDF with program details (e.g., program structure, healthcare benefits, etc) in advance.’ ‘Consider shortening the day, as I feel zoom fatigue is really from the total length of day rather than back to back interviews.’
Individual interviews	‘I felt like I really connected with my interviewers.’ ‘Was able to switch between interviews seamlessly and had good 5 minute and 1 minute warnings before the interviewed finished.’ ‘I liked the standard questions with the program director, it made it feel like he was trying to get to know me as a person.’	‘20 min for interviews felt short, especially with the hard cutoff time. Maybe 25?’ ‘I do not like that the interview rooms timed out automatically. This led to awkward ends to the conversations.’
Interactions with current fellows	‘It was helpful to have multiple opportunities to speak with fellows.’ ‘The strongest aspect of the interview day was the opportunity to chat one-on-one with a current fellow (i.e., a chief fellow sent an introductory email connecting us with another fellow in advance so we could talk by phone). Creating an opportunity for one-on-one private conversation with a current fellow is one of the most impactful things I think a program can do for virtual recruiting.’	‘I would have liked more one-on-one or small group time with the fellows. It is hard to ask questions in large groups.’ ‘I would have liked to talk to fellows who have children (did not get this opportunity over zoom).’
Evaluating educational components	‘Good description of the breadth of research opportunities.’	‘It would have been nice to attend a didactic session, but I don’t think this is compulsory.’
Evaluating program culture	‘They let us sit in on the working groups involving the fellows and faculty, which did give a sense of the culture.’	‘I would have liked to have the opportunity to get a feel for the intangibles of working at this program; specifically, an impression on how much people enjoyed coming to work every day and relationships between colleagues.’ ‘It was harder to get a general sense of what sort of interpersonal interactions exist in the department, sense of camaraderie among the fellows, and what sort of physical presence the fellowship has in the hospital.’
Evaluating the hospital/surrounding city	‘Enjoyed the attempt to do a tour over video.’	‘Hospital set up and facilities where not really shown on interview day. It doesn’t need to take a lot of time, but a little bit of a visual would be useful.’ ‘Could consider a zoom based walkthrough of the various facilities to simulate in-person experience (cafeteria, work rooms, hospital entry shots, etc).’ ‘Hard to assess distance between hospitals and experience of living in the city.’

^a^Shown are representative quotations from applicants responding to the open-ended items: ‘What were the overall strengths and weaknesses/areas from improvement of the UCSF virtual interview day experience?’, organized by themes.

**Table 3.** Recommended considerations for structuring an effective virtual interview day

Domain	Recommended considerations
Structure/organization of the virtual interview day	Recommend that the interview day is long enough to convey the information needed but ideally <7 hours to prevent fatigue, with adequate breaks (3–5) included Provide contact information for the program director or an administrator in case technical issues arise Create meal breaks just for eating, being sensitive to different time zones Consider emailing or mailing overview materials to applicants in advance
Individual interviews	Recommend <30 minute individual interviews
Interactions with current fellows	Recommend sufficient interactions between applicants and fellows, ideally in small groups or one-on-one Consider offering more than one opportunity for applicants to meet fellows (e.g., a virtual social lunch and a panel Q + A or teaching conference) Consider pairing applicants with current fellows via email in advance to provide the opportunity for one-on-one connection outside the virtual interview day, if desired
Evaluating educational components	Create an overview presentation to highlight the educational components of the program, ideally given in real-time (rather than pre-recorded) at the beginning of the virtual interview day
Evaluating program culture	Consider allowing applicants to attend a virtual teaching conference/didactic, particularly those that allow applicants to observe fellow/faculty interaction Directly address sensitive subjects, such as the experience having a family/balancing family obligations and the culture of DEI at the program (e.g., with a planned Q + A session)
Evaluating the hospital/surrounding city	Include a virtual hospital tour, either pre-recorded or in real time (e.g., hospital entrance, fellow work-rooms, training facilities, etc.) Highlight elements of the surrounding city, including where most fellows live and cultural/outdoor opportunities in the surrounding area

### Structure of the virtual interview day

**Figure 1.**
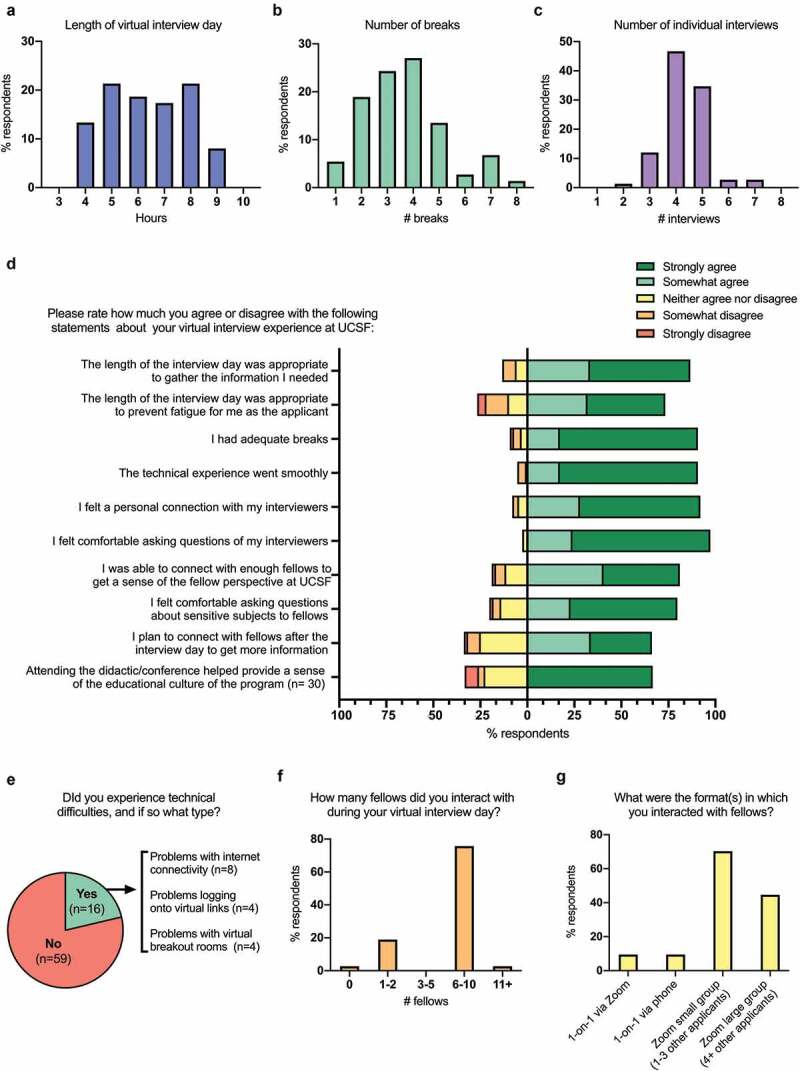
Features of the virtual interview day

Survey respondents reported the virtual interview day lasted an average of 6.4 hours (Figure 1A) with an average of 3.7 breaks (Figure 1B) and 4.3 individual interviews (Figure 1C). Most applicants agreed that the virtual interview day was long enough to gather the necessary information (n = 65, 86.7%) but short enough to prevent fatigue (n = 55, 73.3%) (Figure 1D). The respondents who indicated that they felt fatigued reported a longer average interview day (7.4 hours) than respondents who did not report fatigue (6.0 hours) (p < 0.01). Most respondents agreed that there were adequate breaks (n = 68, 90.7%, Figure 1D). Respondents who indicated that there were an inadequate breaks reported fewer average breaks (2.3 breaks) than respondents who felt that the number of breaks was adequate (4.1 breaks) (p = 0.03). 16 out of 75 applicants (21.3%) reported technical difficulties during the virtual interview day (Figure 1E). Most technical issues were resolved in less than 10 minutes (n = 15, 93.8%). Despite these technical issues, almost all applicants agreed that the technical experience went smoothly (n = 71, 94.7%, Figure 1D).

Survey respondents reported about features of the virtual interview day including: **(A)** the length of the interview day (hours), **(B)** the number of breaks, and (**C)** the number of individual interviews. (**D)** The distributions of survey responses to Likert items about characteristics of the virtual interview day are shown here. Responses to Likert items to the right of the vertical baseline (0% axis) show the percentage of respondents who answered ‘strongly agree’ (dark green) or ‘somewhat agree’ (light green) to the statement shown. Responses to the left of the vertical baseline show the percentage of respondents who answered ‘neither agree nor disagree’ (yellow), ‘somewhat disagree’ (orange), or ‘strongly disagree’ (red) to the statement shown. Survey respondents reported about the (**E**) incidence and types of technical difficulties, (**F**) the number of fellows that they interacted with during the virtual interview day, and (**G**) the format(s) in which they interacted with fellows.

### Individual interviews

Most survey respondents reported that their average individual interview lasted 16–30 minutes (n = 68, 90.7%); a minority reported a 31–45 minute average interview time (n = 7, 9.3%). Most applicants agreed that they felt a personal connection with their interviewers (n = 69, 92.0%) and felt comfortable asking questions (n = 73, 97.3%) (Figure 1D). Of note, there was no statistically significant difference in the number of respondents who agreed that they felt a personal connection with their interviewer or felt comfortable asking questions based on gender, year in training (R2/R3 vs. post-residency), or UIM status (Supplementary Table 4).

### Interactions with fellows

Most respondents reported that they interacted with 6–10 fellows during their virtual interview day (n = 56, 75.7%, Figure 1F). Most fellow interactions occurred during the virtual interview day (n = 68, 91.9%) and/or the night prior (n = 11, 14.9%). Respondents connected with fellows in virtual large groups with 4+ other applicants (n = 33, 44.6%), virtual small groups with 1–3 other applicants (n = 52, 70.3%), one-on-one via a virtual platform (n = 7, 9.5%) and/or one-on-one via telephone (n = 7, 9.5%) (Figure 1G). Most respondents agreed that they had an opportunity to meet enough fellows to get a sense of the fellow perspective (n = 60, 81.1%, Figure 1D). Most respondents agreed that they felt comfortable asking fellows about ‘sensitive subjects’ (e.g., having a family, being a UIM trainee) (n = 59, 79.7%, Figure 1D), and responses to this question did not differ based on gender, year in training (R2/R3 vs. post-residency), or UIM status (Supplementary Table 4).

### Attending a virtual educational conference

30 applicants reported that they attended an educational conference during the virtual interview day (41.7%) and 8 indicated that they were given information about how to access a conference at a later time (11.1%). Of the latter group, 4 planned to attend at a later time (50.0%) and 4 did not (50.0%). Of the 30 applicants who attended during the virtual interview day, two-thirds agreed that attending the virtual conference helped provide a sense of the program’s educational culture (n = 20, 66.7%, Figure 1D).

### Assessing educational opportunities

**Figure 2.**
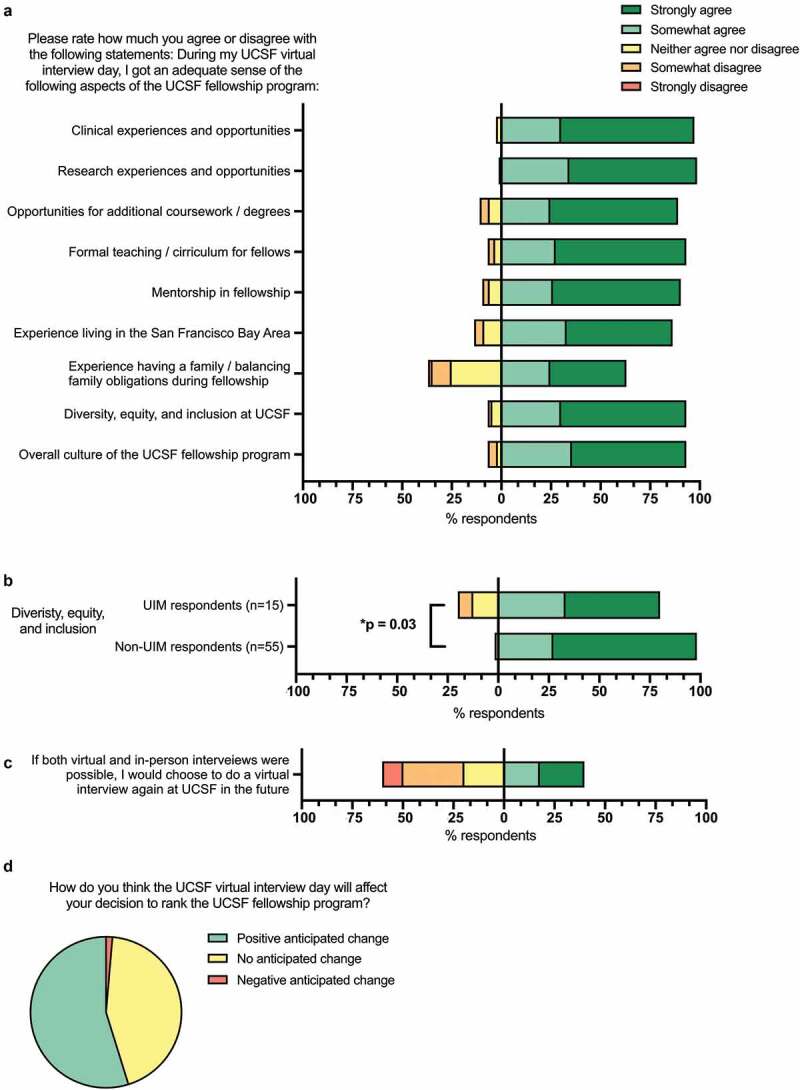
Ability to assess the fellowship components and culture, and overall assessment

Most respondents agreed that the virtual interview day gave them an adequate sense of the educational opportunities at the fellowship program, including the clinical experiences (n = 71, 97.3%), research experiences/opportunities (n = 72, 98.6%), opportunities for additional coursework/degrees (n = 65, 89.0%), formal teaching/curriculum (n = 68, 93.2%), and mentorship (n = 66, 90.4%) (Figure 2A).

(**A**) The distributions of applicant responses to Likert items about their ability to adequately evaluate components of the fellowship program through their virtual interview experience are shown here. Responses to Likert items to the right of the vertical baseline (0% axis) show the percentage of respondents who answered ‘strongly agree’ (dark green) or ‘somewhat agree’ (light green) to the statement shown. Responses to the left of the vertical baseline show the percentage of respondents who answered ‘neither agree nor disagree’ (yellow), ‘somewhat disagree’ (orange), or ‘strongly disagree’ (red) to the statement shown. (**B**) Applicant responses about their ability to adequately assess diversity, equity, and inclusion (DEI) by under-represented in medicine (UIM) status. (**C**) Applicant responses about whether they would choose to interview virtually again if both virtual and in-person interviews were possible. The same Likert scale and visual representation of the results described in (A) were also used for (B) and (C). (**D**) Applicant responses about how they anticipate the virtual interview day will affect their anticipated rank position of the fellowship program.

### Assessing the surrounding city and fellowship culture

Most applicants agreed that the virtual interview gave them an adequate sense of the fellow experience living in the surrounding city (San Francisco) (n = 63, 86.3%, Figure 2A). Most applicants agreed that they were able to get an adequate sense of the overall culture of the fellowship program based on their virtual interview day (n = 68, 93.2%, Figure 2A). Almost two-thirds of respondents agreed that they were able to adequately assess the experience balancing family obligations during fellowship (n = 46, 63.0%, Figure 2A). Responses to this question did not differ by gender, year in training, or UIM status (Supplementary Table 4). Most respondents agreed that they were able to get an adequate sense of the program’s support for diversity, equity, and inclusion (DEI) (n = 68, 93.2%, Figure 2A). Respondents who identified as UIM were less likely to agree that they had an adequate sense of the support for DEI compared to non-UIM respondents (p = 0.03, Figure 2B, Supplementary Table 4).

### Overall impression

Most survey respondents agreed that they were able to get an adequate sense of the overall culture of the UCSF fellowship program based on their virtual interview experience (n = 68, 93.2%). Responses to this question did not vary based on gender, year in training, UIM status, or interview length (Supplementary Tables 4 and 5). Survey respondents were divided about whether they would choose to interview virtually again if both virtual and in-person interviews were possible: 39.7% agreed that they would choose to interview virtually again (n = 29), 20.5% neither agreed nor disagreed (n = 15), and 50.7% disagreed (n = 37) (Figure 2C). Responses to this question also did not vary based on gender, year in training, or UIM status (Supplementary Table 4).

Most applicants indicated that there were areas that they hoped to explore more fully with an in-person visit (n = 53, 72.6%). Free-text responses to explore these areas fell into four broad categories: the opportunity for in-person fellow interaction (n = 27), the opportunity to observe faculty/fellow interactions (n = 16), the opportunity to tour the hospital (n = 29), and/or the opportunity to visit the host city (n = 15). Respondents were also given the opportunity to highlight overall strengths and weaknesses of the virtual interview day experience using free-text responses, and representative responses are provided in Table 2. The most frequently cited strengths were the strong organization of the virtual interview day (n = 24), the welcoming faculty/fellows (n = 15), and time/money saved (n = 14). The most common areas for improvement included the long length of the virtual interview day (n = 11) and the desire to meet more fellows, particularly in small groups or one-on-one (n = 10). Of note, several comments referred to issues that were not addressed elsewhere in the survey, such as challenges with having a common virtual waiting room and abrupt interview cut offs due to automatic closing of the interview rooms.

Finally, when asked whether the virtual interview day experience impacted their anticipated rank position of the fellowship program, 32 applicants indicated an anticipated positive change (43.8%), 40 indicated no anticipated change (54.8%), and 2 indicated an anticipated negative change (2.7%) (Figure 2D).

## Discussion

Our evaluation of virtual interviews demonstrated that this format can be effective way for applicants to evaluate fellowship training programs. Strengths of the virtual interview days included adequate length, number of breaks, organization/technical delivery, connection with interviewers, and the ability to assess the educational and cultural components of the program. There were also opportunities for improvement, such as the suggestion to shorten the length of the virtual interview day and the desire to meet more fellows, particularly in smaller groups or one-on-one. Based on our quantitative and qualitative survey data, we created a list of considerations for how to best design an effective virtual interview day to meet the needs of the applicant (Table 3).

Unlike a prior study which indicated that the virtual interview experience had a negative impact on applicant’s rank order[10], our survey respondents reported that the virtual interview had a neutral or positive effect on anticipated rank order. This may reflect the fit of the program or personal factors more than the virtual interview itself, and may also be viewed differently during an all-virtual interview year. Interestingly, applicants expressed mixed responses about whether they would choose to interview virtually again if both virtual and in-person interview are offered. It is possible that the perception of virtual interviews will continue to improve as programs make changes and applicants become more familiar with this format. In fact, it may be that virtual interviews actually enhance the interview experience in certain ways (e.g., cost savings, decreased travel time, updated supplementary videos and materials, ability to engage faculty for optimal interview pairings given ease of virtual interviews, etc.). Alternatively, there may be certain features of the in-person experience that cannot be fully captured virtually despite best efforts.

After the COVID-19 pandemic, programs may choose to offer both virtual and in-person interviews to meet the needs of all applicants. In this case, it will be critical to evaluate the experience and outcomes of a hybrid approach to ensure equity[16]. For example, if a program offers virtual interviews and optional in-person visits, the latter component could be coordinated by a faculty/staff member not involved in the applicant selection process and after program rank lists are determined.

This study has a number of notable strengths. First, it is one of the largest surveys of GME virtual interview applicant experiences published to date. Second, the 38-item survey comprehensively evaluates the structure of the virtual day and the ability to evaluate program culture using multiple-question types to collect both quantitative and qualitative data. Of note, responses to the Likert questions sometimes differed from the free-text responses. For example, most fellows reported that they were able to get an adequate sense of the surrounding city, but many fellows still indicated a preference to see the city in person. The detail provided by free-text comments offers an important perspective[17], and may highlight areas that were adequately shown virtually but are still desired areas for improvement. Third, although this was a single-center study, it included applicants from eight subspecialty fellowship programs, each of which had a different format, allowing for comparisons between subspecialties and a more comprehensive picture of applicant experiences. Therefore, the results of this survey are generalizable across most IM subspecialties, and likely to residency programs as well.

This survey also has some limitations. First, the response rate was 30.7% (75/244), so there may have been response bias along applicants who had either a positive or negative experience. However, our qualitative data allowed us to gain a rich understanding of the virtual interview applicant experience, thus bolstering our survey conclusions. The response rate among applicants who identified as females and UIM were lower than the total number of applicants in each demographic group, although some survey respondents preferred not to provide demographic information (Table 1, Supplementary Table 2). Second, we chose to send the survey immediately after each interview day and prior to the Fellowship NRMP Match Day. Although we emphasized that the results of the survey would not be viewed prior to Match Day, it is possible that some applicants did not respond due to concern for lack of anonymity and/or that applicants may have responded more positively. Third, there were more respondents in certain subspecialties, such as hematology/oncology and infectious diseases, so the experiences of applicants in these subspecialties were over-represented. Fourth, we did not ask applicants about their home residency institution or familiarity with the UCSF fellowship program prior to the virtual interview day, so it is possible that applicants who trained at UCSF may have had a better baseline understanding of the fellowship program and city. Finally, since all interviews were conducted virtually this year, there was no in-person interview comparison group. In the future, when both virtual and in-person interviews are possible it will be important to study the unique experiences of each to better compare the interview formats.

## Conclusions

In summary, this study provides a comprehensive assessment of the virtual interview experience from the applicant perspective across eight IM subspecialty fellowship programs at our institution, highlighting strengths of this format and areas for improvement. The findings from this study can inform future virtual interviews for GME training programs, which is particularly relevant in the era of COVID-19 and may continue as a common practice after the pandemic.

## Supplementary Material

Supplemental Material
