# Supplementary material for: A single center evaluation of applicant experiences in virtual interviews across eight internal medicine subspecialty fellowship programs
Source: Med Educ Online. 2021 Jun 30;26(1):1946237. doi: 10.1080/10872981.2021.1946237 (PMC8253192; doi:10.1080/10872981.2021.1946237)
Supplement: Supplemental Material [file ZMEO_A_1946237_SM8773.docx]

**Supplemental Data**

**Supplementary Table 1: Planned structure of virtual interview day by subspecialty fellowship program**

| **Subspecialty fellowship program** | Scheduled time (maximum) | Duration of virtual interview day (hours) (maximum) | Number of interviews/applicant | Individual interview length (min) | Number of breaks  (# breaks) | Opportunity to attend a virtual teaching conference? | Format of fellows interaction |
| --- | --- | --- | --- | --- | --- | --- | --- |
| **Clinical informatics** | 8am-2/3pm | 6-7 | 3-4 | 20 | 3+ | Yes- during the day | Virtual lunch |
| **Endocrinology** | 8:30am-1pm OR 12pm-4:30pm | 4.5 | 5 | 25 | 5+ | Yes | Virtual lunch, individual 1:1 interviews with fellows; separate virtual social event (one for all interviewees at a different time) |
| **Gastroenterology** | 7am-12pm OR 1pm-5pm | 4-5 | 4 | 20 | 3+ | No | Virtual social event night prior |
| **Geriatrics** | 8:30am-4pm | 7.5 | 5 | 30 | 3 | Yes- during the day or another day | Virtual lunch |
| **Hematology/**  **oncology** | 8am-5pm | 9 | 4 | 30 | 4+ | No | Virtual lunch and 1-on-1 via phone |
| **Hospice & palliative med** | 8:30am-2pm | 5.5 | 3 | 30 | 4+ | Yes- another day | Virtual lunch |
| **Infectious diseases** | 7:30am-3pm | 7.5 | 5 | 25 | 3 | Yes- during the day | Virtual lunch |
| **Rheumatology** | 7:45am-2:45pm | 7 | 5 | 30 | 6 | Yes- during the day and another day | Virtual lunch |

**Supplementary Table 2: Survey respondent demographic characteristics and reported features of the virtual interview day by subspecialty fellowship program**

|  | **Number of survey respondents and**  **self-reported demographic characteristics^a^** | | | **Reported features of the**  **virtual interview day^b^** | | |
| --- | --- | --- | --- | --- | --- | --- |
| **Subspecialty fellowship program** | **Survey respondents**  # survey respondents/total applicants who received the survey by subspecialty (% response rate) | **Female**  (# respondents, % survey respondents by subspecialty) | **UIM**  (# respondents, % survey respondents by subspecialty | **Duration of the virtual interview day**  (mean # hours) | **Number of breaks**  (# breaks) | **Technical difficulties**  (# respondents, % subspecialty) |
| **Clinical informatics** | 7/16 (43.8%) | 2 (28.6%) | 0 (0%) | 6.0 | 2.3 | 0 (0%) |
| **Endocrinology** | 7/30 (23.3%) | 6 (85.7%) | 1 (14.3%) | 4.6 | 4.1 | 1(14.3%) |
| **Gastroenterology** | 7/45 (15.5%) | 2 (28.6%) | 1 (14.3%) | 4.7 | 3.1 | 0 (0%) |
| **Geriatrics** | 4/28 (14.3%) | 1 (25.0%) | 0 (0%) | 8.5 | 3.8 | 1 (25.0%) |
| **Hematology/oncology** | 22/50 (44.0%) | 8 (36.4%) | 5 (22.7%) | 6.6 | 3.0 | 6 (27.3%) |
| **Hospice & palliative med** | 7/22 (31.8%) | 7 (100%) | 3 (42.9%) | 6 | 4.3 | 2 (28.6%) |
| **Infectious diseases** | 14/35 (40.0%) | 6 (42.9%) | 4 (28.6%) | 7.3 | 4.9 | 5 (35.7%) |
| **Rheumatology** | 2/18 (11.1%) | 1 (50.0%) | 1 (50.0%) | 8.5 | 3 | 1 (50%) |

^a^Applicants self-reported demographic characteristics. Of note, the percentages of respondents do not reflect the overall demographics of applicants interviewed at the UCSF subspecialty fellowship programs, but just the respondents who completed this survey

^b^Reported features of the virtual interview day from the applicant perspective

Abbreviations: Under-represented in medicine (UIM)

**Supplementary Table 3: Demographic features of survey respondents vs. all applicants**

| **Demographic feature** | Survey respondents (N=75)  n, % survey respondents^a^ | Number of applicants who received the survey^b^ (N=244)  n, % total applicants |
| --- | --- | --- |
| **Female** | 33 (44.0%) | 138 (56.6%) |
| **UIM** | 15 (20.0%) | 64 (26.2%) |

^a^Survey respondents who indicated “prefer not to answer” or did not provide a response are not included as either female or UIM.

^b^Total number of applicants from all eight fellowship programs who received the survey

Abbreviations: Under-represented in medicine (UIM)

**Supplementary Table 4: Differences in survey responses based on gender, year in training, and UIM status**

|  | **Gender^a^** | | | **Year in training^b^** | | | **UIM^c^** | | |
| --- | --- | --- | --- | --- | --- | --- | --- | --- | --- |
| **Agreement with the following statements:^d^** | **Female** | **Male** | **P-value^e^** | **R2/R3** | **Post-residency** | **P-value^e^** | **UIM** | **Not UIM** | **P-value^e^** |
| I felt a personal connection with my interviewer | 30/30 (100%) | 30/35 (85.7%) | 0.06 | 47/49 (95.9%) | 17/19 (89.5%) | 0.31 | 15/15 (100%) | 46/51 (90.2%) | 0.58 |
| I felt comfortable asking questions of my interviewer | 30/30 (100%) | 33/35 (94.0%) | 0.50 | 48/49 (98.0%) | 19/19 (100%) | >0.99 | 15/15 (100%) | 49/51 (96.1%) | >0.99 |
| I felt comfortable asking questions about sensitive subjects to fellows (e.g. having a family, being UIM at the fellowship, etc) | 24/30 (80%) | 29/35 (82.9%) | >0.99 | 41/49 (83.7%) | 13/19 (68.4%) | 0.19 | 12/15 (80.0%) | 40/51 (78.4%) | >0.99 |
| I got an adequate sense of the following aspects of the UCSF fellowship program: Having a family / balancing family obligations during fellowship | 19/30 (63.3%) | 22/35 (62.9%) | >0.99 | 34/49 (69.4%) | 9/19 (47.4%) | 0.10 | 9/15 (60.0%) | 40/51 (78.4%) | 0.19 |
| I got an adequate sense of the following aspects of the UCSF fellowship program: Diversity, equity, and inclusion | 27/30 (90.0%) | 35/35 (100%) | 0.09 | 46/49 (93.9%) | 18/19 (94.7%) | >0.99 | 12/15 (80.0%) | 50/51 (98.0%) | **0.03** |
| I got an adequate sense of the following aspects of the UCSF fellowship program: The overall culture of the UCSF Fellowship program | 27/30 (90.0%) | 33/35 (94.3%) | 0.66 | 47/49 (95.9%) | 16/19 (84.2%) | 0.13 | 15/15 (100%) | 46/51 (90.2%) | 0.58 |
| If both virtual and in-person interviews were possible, I would choose to do a virtual interview at UCSF again in the future | 14/30 (46.7%) | 13/35 (37.1%) | 0.46 | 20/49 (40.8%) | 7/19 (36.8%) | >0.99 | 8/15 (53.3%) | 20/51 (39.2%) | 0.38 |

^a^ Self-reported gender as female or male are included here. Survey respondents who indicated “non-binary”, “prefer not to answer” or who did not respond to the demographic data are not included here.

^b^ Self-reported year in training. Respondents who indicated that they were in their second or third year of residency training are combined and labeled as “R2/R3”. Applicants who indicated that they were in their first year post-residency or beyond were combined and labeled as “Post-residency”.

^c^ Self-reported under-represented in medicine (UIM). See methods section for definition and inclusion criteria for UIM

^d^ Respondents who answered “strongly agree” and “somewhat agree” are considered favorable responses and combined as “agreement” here; “neither agree nor disagree”, “somewhat disagree”, and “strongly disagree” are considered unfavorable responses.

^e^ Two-sided Fisher’s exact test, p-value

**Supplementary Table 5: Differences in program assessment based on interview length**

|  | **Interview length^a^** | | |
| --- | --- | --- | --- |
| **Agreement with the following statements: ^b^** | ≤**6 hours** | **>6 hours** | **P-value^c^** |
| I got an adequate sense of the following aspects of the UCSF fellowship program: |  |  |  |
| Clinical experiences and opportunities | 36/37 (97.3%) | 34/35 (97.1%) | >0.99 |
| Research experiences and opportunities | 36/37 (97.3%) | 35/35 (100.0%) | >0.99 |
| Opportunities for additional coursework /  degrees | 32/37 (86.5%) | 32/35 (91.4%) | 0.71 |
| Formal teaching / curriculum for fellows | 35/37 (94.6%) | 32/35 (91.4%) | 0.67 |
| Mentorship in fellowship | 33/37 (89.2%) | 32/35 (91.4%) | >0.99 |
| Experience living in the San Francisco Bay  Area | 31/37 (83.8%) | 31/35 (88.6%) | 0.74 |
| Experience having a family / balancing  family obligations during fellowship | 26/37 (70.3%) | 19/35 (54.3%) | 0.22 |
| Diversity, equity, and inclusion | 35/37 (81.1%) | 32/35 (91.4%) | 0.67 |
| The overall culture of the UCSF Fellowship  program | 33/37 (89.2%) | 34/35 (97.1%) | 0.36 |
| If both virtual and in-person interviews were possible, I would choose to do a virtual interview at UCSF again in the future | 15/37 (40.5%) | 14/35 (40.0%) | >0.99 |

^a^ Self-reported interview length.

^b^ Respondents who answered “strongly agree” and “somewhat agree” are considered favorable responses and combined as “agreement” here; “neither agree nor disagree”, “somewhat disagree”, and “strongly disagree” are considered unfavorable responses.

^c^ Two-sided Fisher’s exact test, p-value

**Survey Instrument**

Given the transition to virtual interviews for the 2020-2021 fellowship application season, we are interested in better understanding applicant perceptions, experiences, and preferences about the virtual interview experience. Therefore, we would be grateful if you completed this optional, anonymous survey about your virtual interview day experience at UCSF. **We will not view or analyze any of the survey results until after Match Day (Dec 2, 2020).**

**Technology and interviews**

- Did you encounter any technical difficulties on your UCSF virtual interview day? (yes/no)
- If yes, which of the technical difficulties did you encounter (check all that apply):
- Problems with internet connection / connectivity
- Problems logging onto the Zoom links
- Problems with Zoom breakout rooms or other features
- Other technical issues (please specify) [free text]
- If yes, how quickly were these technical issues resolved?
- <10 minutes
- 11-20 minutes
- 21-30 minutes
- >30 minutes
- Issues was not resolved during the interview day
- How long was your virtual interview day? [slider hours]
- How many individual interviews did you have? [slider number individual interviews]
- How long was each individual interview (on average)?
  - 15 minutes or less
  - 16-30 minutes
  - 31-45 minutes
  - More than 45 minutes
- How many breaks did you have throughout the day? [slider of number of breaks]
- Please rate how much you agree or disagree with the following statements about your UCSF virtual interview day:
- The technological experience of my UCSF interview day went smoothly
- I had adequate breaks throughout the virtual interview day
- The length of the interview day was appropriate to prevent fatigue for me as the applicant
- The length of the interview day was appropriate to gather the information I needed
- I was able to highlight my strengths during my virtual interviews
- I felt a personal connection with my interviewers
- I felt comfortable asking questions of the interviewer
- Strongly agree
- Somewhat agree
- Neither agree nor disagree
- Somewhat disagree
- Strongly disagree

**Connecting with fellows on the virtual interview day**

- How many fellows did you get to speak with during your virtual interview day?
  - None
  - 1-2
  - 3-5
  - 6-10
  - More than 10
- What were the ways in which you connected with fellows? [check all that apply]
  - One-on-one via Zoom
  - In Zoom small groups or breakout rooms with 3 or fewer applicants
  - In Zoom larger groups or breakout rooms with 4 or more applicants
  - Other (please specify) [free text]
- What was the timing of the fellow interaction? [check all that apply]
- Night before the virtual interview day
- During my virtual interview day
- The evening after my virtual interview day
- Another day after my virtual interview day (planned for the future)
- Other (please specify) [free text]
- Please rate how much you agree or disagree with the following statements:
  - I was able to connect with enough fellows to get a sense of the fellow perspective at UCSF
  - I felt comfortable asking questions about sensitive topics to fellows (e.g. having a family, being UIM at this fellowship, etc.)
  - I plan to connect with fellows after the interview day to get more information
- Strongly agree
- Somewhat agree
- Neither agree nor disagree
- Somewhat disagree
- Strongly disagree

**Learning about the fellowship program**

- Please rate how much you agree or disagree with the following statement: During my UCSF virtual interview day, I got an adequate sense of the following aspects of the UCSF fellowship program:
  - Clinical experiences and opportunities
  - Research experiences and opportunities
  - Opportunities for additional coursework / degrees
  - Formal teaching / curriculum for fellows
  - Mentorship In fellowship
  - Experience living in the San Francisco Bay Area
  - Experience having a family / balancing family obligations during fellowship training
  - Diversity, equity, and inclusion at UCSF
  - Overall culture of the fellowship program
- Strongly agree
- Somewhat agree
- Neither agree nor disagree
- Somewhat disagree
- Strongly disagree
- Were you given the opportunity to attend a fellowship didactic session or conference/grand rounds during the interview day?
- Yes, I attended during the interview day
- Yes, I was given a link to watch at a different time
- No, this was not offered
- If given a link to watch a conference at a different time, will you plan to attend?
- Yes, I will plan to attend
- No, I will not attend because it won’t change my impression of the program
- No, I will not attend because it conflicts with my residency/clinical schedule
- If yes, please rate how much you agree or disagree with the following statement: Attending the didactic/conference helped provide a sense of the educational culture of the program.
- Strongly agree
- Somewhat agree
- Neither agree nor disagree
- Somewhat disagree
- Strongly disagree

**Overall perception of the UCSF virtual interview day**

- Please rate how much you agree or disagree with the following statements: If both virtual and in-person interviews were possible, I would choose to do a virtual interview at UCSF again in the future.
- Strongly agree
- Somewhat agree
- Neither agree nor disagree
- Somewhat disagree
- Strongly disagree
- How do you think the UCSF virtual interview day will affect your decision to rank the UCSF program?
- No change in anticipated rank position
- Positive impact in anticipated rank position
- Negative impact in anticipated rank position
- After having experienced the virtual interview day, is there anything you would have hoped to explore more fully with an in-person visit?
  - Yes
  - No
- If yes, what would you have wanted to explore more fully with an in-person visit if it were possible? [free text]
- Overall, what were the strengths of the virtual interview day experience at UCSF? [free text]
- Overall, what were the weaknesses/areas for improvement of the virtual interview day experience at UCSF? [free text]

**Demographic Information**

- Gender
- Female
- Male
- Non-binary
- Prefer to self-describe: ______ [free text]
- Prefer not to answer
- How do you self-identify? (choose all that apply)
- Hispanic, Latino, or of Spanish origin
- American Indian or Alaskan Native
- Asian
- Black or African American
- Native Hawaiian or Pacific Islander
- White
- Other: ______
- Prefer not to answer
- Current year in training or post-training?
- Second year resident (PGY2)
- Third year resident (PGY3)
- One-year post residency
- Two years post residency
- Three or more years post residency
- What specialty are you applying to at UCSF?
  - Addiction Medicine
  - Allergy/Immunology
  - Cardiology
  - Clinical Informatics
  - Endocrinology
  - Gastroenterology
  - Geriatrics
  - Geriatrics and Palliative Combined Fellowship
  - Hematology/Oncology
  - Hospice and Palliative Medicine
  - Infectious Disease
  - Nephrology
  - Pulmonary/Critical Care
  - Rheumatology
